# Incidence and Risk Factors of Orthostatic Hypotension and Postural Tachycardia Following Sedated Colonoscopy: A Prospective Observational Study

**DOI:** 10.3390/diagnostics15233009

**Published:** 2025-11-26

**Authors:** Gülencan Yumuşak Ergin, Mustafa Ergin, Menekşe Özçelik

**Affiliations:** 1 Department of Anesthesiology and Reanimation, Aksaray University Training and Research Hospital, 68000 Aksaray, Turkey; 2 Department of Gastroenterology, Aksaray University Training and Research Hospital, 68000 Aksaray, Turkey; mstfergn@hotmail.com; 3 Department of Anesthesiology and Reanimation, Ankara University Faculty of Medicine, 06620 Ankara, Turkey; mozcelik@ankara.edu.tr

**Keywords:** ambulatory anesthesia, orthostatic hypotension, post-anesthesia care, postural orthostatic tachycardia syndrome

## Abstract

**Background/Objectives**: Colonoscopy, a common outpatient procedure requiring bowel preparation, can lead to dehydration and electrolyte disturbances. Sedation, while improving patient comfort, may exacerbate these effects and contribute to orthostatic hypotension (OH) and postural orthostatic tachycardia syndrome (POTS). This study aimed to determine the prevalence of OH and POTS following sedated colonoscopy and to identify associated risk factors. **Methods**: This prospective observational study included 76 adult patients (ASA I–III) who underwent colonoscopy with fentanyl–propofol sedation between August and November 2024. Blood pressure, heart rate, and orthostatic intolerance (OI) symptoms were assessed before and after mobilization. OH was defined as a systolic blood pressure decrease ≥20 mmHg or diastolic decrease ≥10 mmHg upon standing. POTS was defined as a heart rate increase ≥30 bpm or an absolute heart rate ≥ 120 bpm. Statistical analyses were performed using SPSS 29.0. **Results**: Post-procedural OH and/or POTS occurred in 18 patients (23.7%), and 14 patients (18.4%) reported OI symptoms such as dizziness, nausea, or blurred vision. Symptomatic patients were significantly younger than asymptomatic patients (42.7 ± 15.4 vs. 54 ± 13.9 years, *p* = 0.009), and symptoms were more frequent among females (*p* = 0.046). Preoperative diastolic blood pressure was significantly higher in patients who developed OH (*p* = 0.022), while other hemodynamic and demographic variables showed no significant associations. **Conclusions**: Orthostatic hypotension and postural tachycardia are relatively common after sedated colonoscopy. Younger age and female sex were identified as independent risk factors for OI symptoms, suggesting a possible role of autonomic variability. Routine post-procedure monitoring and assisted mobilization before discharge may improve patient safety and recovery outcomes.

## 1. Introduction

Colonoscopy is among the most frequently performed medical procedures worldwide, and its prevalence is expected to continue rising with the increasing demand for colorectal cancer screening [1]. Although typically performed as a day-case procedure, colonoscopy requires extensive bowel preparation, which distinguishes it from most other outpatient interventions. Standard bowel preparation protocols involve discontinuing fiber-rich foods 48–72 h before the procedure and restricting intake to clear liquids during the final 24 h. This preparation frequently causes osmotic diarrhea and enhanced intestinal peristalsis, potentially leading to hypovolemia and electrolyte disturbances [2].

Certain regimens that require high volumes of clear liquids may result in significant fluid deficits and, in some cases, hyponatremia. In addition, nausea and reduced oral intake are common pre-procedural findings [3]. Beyond these preparatory effects, colonoscopy itself is often associated with abdominal discomfort, bloating, and cramps. Sedation—routinely administered to improve patient comfort and procedural efficiency—can further increase the risk of mild hypotension [4]. When combined with pre-existing dehydration, sedation may delay postoperative recovery by provoking orthostatic intolerance (OI) symptoms [5].

OI symptoms, including dizziness, fatigue, and syncope, are common after anesthesia and are linked to delayed discharge, increased postoperative morbidity, and higher readmission rates. Medications used for premedication, anesthesia, and analgesia can decrease arterial pressure, cerebral blood flow, and oxygenation, thereby predisposing patients to OI [6].

A key mechanism underlying OI is orthostatic hypotension (OH), defined as a sustained reduction in systolic blood pressure (SBP) ≥ 20 mmHg or diastolic blood pressure (DBP) ≥ 10 mmHg upon standing [7,8]. Clinically, OH presents with nausea, vomiting, dizziness, or syncope, all of which may complicate early mobilization and discharge [9]. Despite being well recognized in postoperative care, the mechanisms and management strategies of OH remain poorly characterized. Postural orthostatic tachycardia syndrome (POTS)—also referred to as postural autonomic tachycardia or chronic/idiopathic OI—is another form of orthostatic dysfunction, predominantly affecting younger individuals. It is characterized by an increase in heart rate ≥ 30 beats per minute or an absolute rate ≥ 120 beats per minute upon standing, without a corresponding drop in blood pressure. POTS is typically accompanied by fatigue, dizziness, cognitive difficulties, and reduced exercise tolerance, although its underlying pathophysiology remains unclear [10].

While the prevalence and risk factors of OH and POTS have been extensively studied in the context of general anesthesia, data on their occurrence following outpatient procedures performed under sedation are limited. This study aims to fill that gap by determining the prevalence of OH and POTS and identifying related risk factors in patients undergoing sedated colonoscopy. Understanding these factors is essential for improving patient safety, shortening recovery times, and facilitating safe and timely discharge in ambulatory endoscopy units. Although OH and POTS are often described as distinct clinical entities, they share overlapping autonomic and circulatory mechanisms. As highlighted in prior consensus and mechanistic studies [11,12], OH and POTS are overlapping manifestations of orthostatic intolerance rather than entirely distinct entities [13,14,15]. Accordingly, both were analyzed together in this study to provide a comprehensive evaluation of post-sedation hemodynamic instability.

Therefore, evaluating OH and POTS together may provide a more comprehensive understanding of the spectrum of post-sedation orthostatic intolerance and its implications for patient recovery and discharge safety. Both are characterized by transient baroreflex dysfunction, relative hypovolemia, and excessive heart rate response after positional change, which can be exacerbated by sedative agents such as propofol and opioids. Recent evidence suggests that propofol attenuates sympathetic tone and blunts vascular resistance, while opioids may potentiate vagal activity, jointly increasing susceptibility to orthostatic hemodynamic shifts even after minor procedures. Consistent with these findings, a recent meta-analysis demonstrated that hypotension occurs in approximately one-third of patients receiving propofol sedation for colonoscopies [16,17].

We hypothesized that a measurable proportion of patients undergoing sedated colonoscopy experience post-procedural orthostatic hypotension and/or postural tachycardia, and that pre-procedural diastolic blood pressure, fasting duration, and patient sex may independently predict these outcomes.

## 2. Materials and Methods

### 2.1. Study Design, Ethics Approval, and Consent to Participate

This single-center prospective study was conducted between August and November 2024 in the endoscopy unit of Aksaray University Training and Research Hospital. Ethical approval was obtained from the Human Ethics Review Committee of Aksaray University Hospital (approval number: 2023/22-03, date: 23 November 2023), and the study was registered at ClinicalTrials.gov (ID: NCT06439498, date: 28 May 2024). The study was performed in accordance with the principles of the Declaration of Helsinki and was approved by the Institutional Ethics Committee. Written informed consent was obtained from all participants prior to enrollment.

### 2.2. Participants

**Figure 1:**
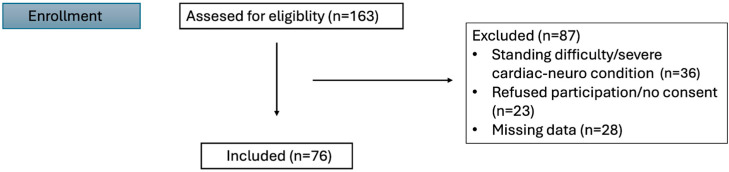
Flowchart of the study.

A total of 76 adult patients aged 18–80 years, classified as ASA I–III, who underwent colonoscopy performed by a gastroenterologist, were enrolled. All patients underwent bowel preparation with a polyethylene glycol-based regimen according to the institutional protocol. All patients underwent routine pre-procedural laboratory evaluation, including complete blood count and serum electrolyte analysis, as part of the standard anesthesia assessment. Patients presenting with anemia, electrolyte imbalance, or any clinically significant abnormality were excluded to ensure hemodynamic stability and to minimize potential confounding effects on study outcomes. Patients with difficulty standing, or those with severe cardiac or neurological disorders, were excluded. Individuals who reported symptoms suggestive of OH—such as dizziness or lightheadedness upon standing—or with a previous history of orthostatic hypotension or recurrent postural dizziness were also excluded, as were patients who declined to provide written informed consent (Figure 1).

### 2.3. Anesthesia Management

All patients underwent routine preoperative anesthesia evaluation. Fasting duration and bowel preparation medications were documented before the procedure. Standard ASA monitoring included non-invasive blood pressure (SBP, DBP, and mean arterial pressure, heart rate, oxygen saturation, and continuous electrocardiography. Supplemental oxygen was delivered via nasal catheter at 6 L/min. Blood pressure was first measured preoperatively and immediately after induction, followed by automatic recordings at 3-min intervals throughout the procedure. After completion of colonoscopy, an additional measurement was taken upon arrival to the PACU and repeated at 3-min intervals during orthostatic testing.

Anesthesia was induced with intravenous fentanyl (1 µg/kg) and propofol (1–2 mg/kg). The induction dose was administered slowly within 60 s or until the loss of the corneal-palpebral reflex and eyelid drooping were observed. When patients exhibited signs of discomfort (motor or vocal reaction, tachycardia, and/or hypertension), a maintenance bolus of propofol (0.5 mg/kg) was administered as needed.

Personalized fluid therapy was not implemented; instead, all patients received 250 mL of 0.9% isotonic sodium chloride solution during the procedure. This fixed-volume regimen was chosen to minimize inter-operator variability in a high-volume endoscopy unit where different anesthesiologists alternate daily, and to maintain procedural consistency given the short duration of sedated colonoscopy. All colonoscopies were performed by a single experienced gastroenterologist. Vital signs and medications administered before, during, and after the procedure were recorded. Hypotension was defined as a reduction greater than 20% from pre-anesthetic baseline values. Patients who developed hypotension during or after the procedure received 5 mg of ephedrine as intermittent bolus doses. Procedure duration was also recorded.

### 2.4. Post-Procedure Management

After completion of colonoscopy, patients were transferred to the Post-Anesthesia Care Unit (PACU). Those achieving a Modified Aldrete Score ≥ 9 were considered eligible for discharge [18]. No oral fluids were allowed between the end of the procedure and completion of orthostatic testing. Orthostatic vital signs were recorded after full recovery from sedation, verified by an Aldrete recovery score ≥ 9, approximately 15–20 min following discontinuation of propofol infusion. This interval ensured that residual sedative effects were minimal and did not influence hemodynamic responses during orthostatic testing.

Patients who met discharge criteria were first observed in a seated position for 3 min, with continuous monitoring of vital signs. If no hypotension or OI symptoms occurred, they were assisted to stand. Vital signs were reassessed during the first 3 min after standing. OI was defined as intolerable dizziness, nausea, vomiting, a sensation of warmth, or blurred vision during mobilization [8,19]. OH was defined as a decrease in SBP ≥ 20 mmHg or DBP ≥ 10 mmHg within 3 min of sitting or standing. Patients meeting the diagnostic criteria for POTS were analyzed within the OH group [8,19]. POTS was defined as a heart rate ≥ 120 beats/min or an increase ≥ 30 beats/min when transitioning from supine to standing [10]. Patients who developed OI symptoms earlier during testing were immediately returned to the prior position.

After completing the monitored phase in the PACU, patients were transferred to a separate recovery area without continuous monitoring, where they remained under nurse supervision for approximately one hour before final discharge from the endoscopy unit.

### 2.5. Outcome Measures

The primary outcome was the incidence of OH and postural tachycardia following colonoscopy under sedoanalgesia. The secondary outcome was the identification of demographic characteristics and potential risk factors associated with the development of OH and POTS.

### 2.6. Statistical Analysis

Categorical variables were expressed as numbers (n) and percentages (%). Continuous variables were presented as the mean ± standard deviation (SD) for normally distributed data and as median (min–max) for non-normally distributed data. Comparisons between categorical variables were performed using the Chi-square or Fisher’s exact test. Continuous variables between two independent groups were compared using Student’s *t*-test for normally distributed data and the Mann–Whitney *U* test otherwise. The type I error margin (α) was set at 0.05. All statistical analyses were performed using SPSS version 29.0 (IBM Corp., Armonk, NY, USA).

Because of the relatively small number of orthostatic events observed (n = 18), separate analyses for isolated OH and POTS were not statistically feasible. To preserve reliability, these conditions were evaluated together as a combined outcome (OH ± POTS), reflecting the overall presence of post-procedural orthostatic intolerance. Given the limited number of events per variable, multivariable logistic regression was not conducted to avoid model overfitting.

In addition to reporting precision through 95% confidence intervals for the primary outcome, a post hoc power analysis was performed using a two-sided one-sample proportion test (α = 0.05). For interpretability in a single-arm design, a reference incidence (p_0_ = 12%) was selected as a conservative lower-bound benchmark derived from previously reported ranges for postoperative orthostatic events. With n = 76, the achieved power against p_0_ = 0.12 was approximately 0.88.

## 3. Results

A total of 76 patients underwent colonoscopy under sedoanalgesia. The demographic characteristics of the study population are summarized in Table 1. The analysis examined whether coronary artery disease, diabetes mellitus, and hypertension were significant risk factors for postoperative orthostatic complications. Among hypertensive patients, antihypertensive medications were categorized as beta-blockers, angiotensin-converting enzyme inhibitors (ACEi) or angiotensin receptor blockers (ARBs), calcium channel blockers (CCBs), and diuretics.

Postoperative OH was observed in 14 patients (18.4%). Of these, 5 patients also experienced tachycardia, while the total number of patients with postural orthostatic tachycardia was 9 (11.8%). Patients with postural orthostatic tachycardia were evaluated together with those who had OH, resulting in a combined group of 18 patients (23.7%) classified as the OH group. The observed incidence of orthostatic hypotension and/or postural tachycardia was 23.7% (95% CI, 14.1–33.3%). In a post hoc analysis versus a reference incidence of p_0_ = 12%, the achieved power was approximately 0.88 (two-sided, α = 0.05). Assuming a reference incidence (p_0_) of 12%, the minimal detectable incidence corresponding to 80% power was 22.4%, indicating that the sample size was sufficient to detect clinically meaningful differences.

OI symptoms were recorded in 14 patients during the sitting or standing assessments. Among these, 3 patients exhibited hypotension, and 5 patients demonstrated tachycardia without hypotension. No significant changes in vital parameters were detected in the remaining symptomatic patients. Hypotension occurred in 3 patients while seated and in 11 patients upon standing. The majority of patients with measured hypotension did not report associated symptoms.

The mean time from the completion of colonoscopy to achieving a Modified Aldrete Score ≥ 9 was 13 ± 4 min (range: 10–20 min). The recovery time did not differ significantly between patients who developed orthostatic changes and those who did not (13.4 ± 3.8 min vs. 12.9 ± 4.1 min, *p* = 0.46).

The relationships between potential risk factors and OH are presented in Table 2, and those between risk factors and OI symptoms are shown in Table 3. The association between preoperative blood pressure and the development of hypotension was analyzed separately for orthostatic and intraoperative hypotension (Table 4). Patients who developed OH had significantly higher preoperative DBP than those who did not (*p* = 0.022). Although the mean difference in diastolic blood pressure was modest, the effect size (Cohen’s *d* = 0.34) indicated a small but measurable difference between groups. In contrast, no significant association was observed between preoperative systolic or diastolic blood pressure and intraoperative hypotension (*p* = 0.646 and *p* = 0.581, respectively).

There were no statistically significant differences between the groups in terms of SBP, heart rate, or other demographic and clinical parameters. The use of antihypertensive agents, including beta-blockers, ACEi, ARBs, CCBs, and diuretics, was not associated with the occurrence of OH (*p* > 0.05 for all comparisons).

A significant difference in age was observed between the symptomatic and asymptomatic groups (*p* = 0.009), with a lower mean age in the symptomatic group. Orthostatic symptoms were also more frequent among female patients compared with males (*p* = 0.046).

## 4. Discussion

In this study, postoperative OH and/or POTS occurred in 23.7% of patients undergoing colonoscopy under sedoanalgesia, while OI symptoms were observed in 18.4% of cases. Reported incidences of OH and OI in the literature vary widely depending on the type of surgery. Postoperative OH and OI have been more frequently documented after major surgical procedures. For example, OH was identified in 38.6% of patients during early mobilization following cardiothoracic and abdominal surgery [20]. In contrast, after minor surgery, OH was reported in 49% of cases at 15 min and 41% at 45 min postoperatively [21]. Retrospective studies have reported OI incidences ranging from 12% to 60% [22,23,24,25]. The frequency of OI symptoms after open prostatectomy has been reported as 50% at 8 h and 12% at 22 h postoperatively [18]. In total hip arthroplasty, OI occurred in 42% of patients at 6 h and 19% at 24 h after surgery [22]. Müller et al. examined OH and OI in patients undergoing breast surgery and noted that tissue oxygenation was largely maintained during hypotensive episodes. Differences in fluid and pain management strategies among these studies appear to account for variability in reported rates, as optimized management was associated with lower incidences of OH [26].

Studies conducted after day-case surgery and minor procedures have also identified transient orthostatic changes during early mobilization, with symptoms typically resolving within minutes [21,26]. Similarly to these findings, the majority of our patients experienced mild, short-lived hemodynamic alterations that did not delay discharge. However, our results differ from inpatient surgical series, where prolonged anesthesia and higher opioid exposure were associated with more persistent hypotension and delayed recovery [27]. To our knowledge, however, no previous study has specifically investigated the incidence of orthostatic hypotension following colonoscopy, highlighting the novelty of our findings.

Given that the lower bound of previously reported ranges approximates 12%, adopting p_0_ = 12% as the reference provided an achieved power of 0.88, which indicates that the sample size was sufficient to detect clinically meaningful differences in the primary outcome.

Identifying risk factors for OH and OI remains essential for prevention. Because of the limited number of orthostatic events, separate analyses for OH and POTS were not statistically feasible. Therefore, these entities were evaluated together as a combined outcome (OH ± POTS) to ensure statistical reliability. Future studies with larger sample sizes are warranted to investigate potential differences between the two conditions. In this study, only elevated preoperative DBP was associated with the occurrence of OH. No significant differences were observed between groups in SBP, heart rate, or other demographic or clinical parameters. Although previous research has suggested that higher baseline blood pressure may predispose patients to perioperative hypotension and serve as a potential risk factor for post-induction hypotension, the current findings did not demonstrate a statistically significant relationship between preoperative hypertension and reductions in blood pressure after induction or during intraoperative monitoring [28,29]. The small effect size of the observed DBP difference (Cohen’s *d* = 0.34) further supports that this finding, while statistically evident, may have limited clinical relevance.

The continued use of antihypertensive medications, which were not discontinued before the procedure, may also contribute to these conditions [6]. However, the exact timing of medication intake on the day of colonoscopy was not consistently documented, limiting evaluation of their immediate hemodynamic effects. Earlier studies have indicated that advanced age increases the risk of early postoperative OH and OI [22,30].

In the present cohort, patients exhibiting orthostatic intolerance (OI) symptoms were on average younger than those without symptoms. The higher frequency of orthostatic symptoms among younger and female patients may reflect intrinsic differences in autonomic regulation and vascular reactivity. Younger individuals typically display greater heart rate variability and more pronounced sympathetic activation in response to postural stress, which can predispose them to transient tachycardic or hypotensive episodes under physiological challenges [24]. Although population-based studies have traditionally associated orthostatic intolerance with older age [31,32], physiological data suggest that aging affects the autonomic nervous system at multiple levels. With increasing age, sympathetic activity indices rise, while parasympathetic activity and baroreflex sensitivity decline, leading to blunted cardiovascular responses and reduced perception of orthostatic symptoms. In contrast, younger individuals with greater autonomic variability and stronger sympathetic reactivity may experience more pronounced orthostatic symptoms despite comparable blood pressure changes [33].

In women, hormonal fluctuations—particularly cyclic variations in estrogen and progesterone—affect vascular tone and plasma volume regulation, potentially increasing susceptibility to postural hemodynamic instability. These physiological mechanisms likely contribute to the greater symptom burden observed among female patients. Consistent with prior studies, young women have been reported to exhibit higher rates of orthostatic intolerance, possibly due to enhanced vagal responsiveness and lower peripheral vascular resistance [13,14,15]. Collectively, these findings suggest that autonomic and vascular differences associated with age and sex play a central role in post-sedation orthostatic responses. No additional risk factors for OI were identified in this study.

The mean pre-procedural fasting duration was approximately 15 h. Although patients were instructed to consume clear fluids until two hours before the procedure, extended fasting periods were observed. This may have been influenced by limited preoperative counseling and inconsistent reinforcement of fluid intake instructions. Following the completion of this study, the nursing staff responsible for preoperative education received targeted feedback regarding fasting duration. Previous studies have also reported that preoperative fasting times frequently exceed recommended guideline limits [34]. Future investigations could assess the effectiveness of such educational interventions and their integration into routine clinical practice.

The pharmacodynamic properties of propofol and fentanyl may have contributed to the transient orthostatic responses observed in this cohort. Propofol decreases systemic vascular resistance through direct smooth muscle relaxation and attenuation of sympathetic tone, while fentanyl can enhance vagal activity and blunt baroreceptor-mediated compensatory responses [35]. The combined use of these agents during short procedures may temporarily impair autonomic compensation during positional change, even after apparent clinical recovery. From a practical standpoint, these findings highlight the importance of supervised standing and hemodynamic monitoring in the post-anesthesia care unit. Implementing structured post-sedation observation protocols and verifying hemodynamic stability before discharge may help reduce unrecognized orthostatic events and improve patient safety in high-volume endoscopy settings.

Because not all patients with hypotension exhibited symptoms, continuous monitoring in the PACU and supervised standing are recommended even when patients appear clinically stable. In patients with OI, hypotension and tachycardia are not the sole contributors to intolerance symptoms. Consequently, even when discharge is based on the Modified Aldrete scoring system, additional symptom assessment should be incorporated. In high-throughput clinical settings such as endoscopy units, patients should remain under professional supervision for a defined observation period, even if formal discharge criteria are met [36]. Although colonoscopy is considered a minor procedure, its requirement for bowel preparation differentiates it from other outpatient interventions. Given the high daily procedural volume, ensuring safe and timely discharge is particularly important.

### Limitations

This study has several limitations. First, pre-procedural orthostatic testing was not performed, which restricts the ability to determine whether orthostatic hypotension or postural tachycardia observed post-procedure represented new-onset or pre-existing conditions. Therefore, the reported incidence should be interpreted with caution, as some patients might have had an underlying predisposition to orthostatic intolerance. In addition, the single-center design and limited sample size may reduce generalizability. To minimize inclusion of patients with preexisting autonomic dysfunction, individuals with a history of orthostatic symptoms, such as dizziness or syncope, were excluded. Future studies including baseline orthostatic evaluation could help better delineate procedure-related hemodynamic responses.

Although baseline laboratory tests were performed during preoperative anesthesia evaluation, additional laboratory measurements were not repeated after bowel preparation or post-procedure due to institutional workflow constraints. Therefore, potential transient electrolyte changes could not be reassessed.

Another limitation concerns the assessment of discharge and post-discharge monitoring. In our unit, patients underwent a two-step recovery process: an initial monitored phase in the PACU until achieving a Modified Aldrete Score ≥ 9, followed by a nurse-supervised observation period lasting approximately one hour before discharge. Recovery time was quantified as time to discharge readiness in the monitored PACU, as this interval was precisely time-stamped for all participants. However, patients were not followed up after leaving the recovery unit, which may have led to underrecognition of delayed orthostatic symptoms. Future studies could address this limitation by incorporating short-term post-discharge observation or telephone follow-up to detect late-onset orthostatic intolerance events.

Additionally, a multivariate logistic regression analysis was not performed because of the limited number of outcome events relative to the sample size, which could result in model overfitting and unreliable estimates. Therefore, only univariate associations were examined, and this limitation was acknowledged.

One other limitation of the present study is the lack of individualized fluid therapy. The choice of a fixed 250 mL isotonic saline bolus reflected an effort to minimize inter-operator and procedural variability in a high-volume endoscopy unit where different anesthesiologists alternate daily. This conservative regimen is consistent with evidence suggesting that modest fluid administration during routine colonoscopy has limited hemodynamic benefit. According to previous studies, no difference in the incidence of hypotension was observed between patients receiving intravenous fluids (mean 325 mL) and those who did not [37]. Likewise, a randomized controlled trial reported that an average infusion volume of 359 mL offered no measurable clinical advantage while increasing procedural cost [38]. However, in the latter study, patients were allowed to take oral fluids up to two hours before the procedure, whereas in our cohort prolonged fasting was common; therefore, the uniform 250 mL infusion may have served to partially compensate for preoperative fluid restriction. Therefore, our standardized approach was intentionally designed to ensure procedural uniformity while recognizing that the absence of individualized fluid titration remains a methodological limitation.

## 5. Conclusions

Orthostatic hypotension and postural tachycardia appear to be relatively frequent but generally transient phenomena following sedated colonoscopy. Although colonoscopy is typically considered a low-risk procedure, the combined effects of bowel preparation, fasting, and sedative medications may predispose certain patients—particularly younger individuals—to temporary autonomic imbalance and orthostatic intolerance. The present findings should be interpreted as exploratory and hypothesis-generating. Larger, multicenter studies integrating standardized pre- and post-procedural autonomic evaluation, individualized hydration protocols, and follow-up after discharge are warranted to confirm these associations and to develop preventive strategies that enhance post-procedural safety.

## Figures and Tables

**Table 1:** Baseline characteristics of the patients.

Characteristics	n (%)/Mean ± SD/Median (Min–Max)
Age	51.9 ± 14.7
Sex	
Female	36 (47.4)
Male	40 (52.6)
BMI	27.6 ± 4.7
ASA status	
ASA 1	22 (28.9)
ASA 2	47 (61.8)
ASA 3	7 (9.2)
CAD	10 (13.2)
DM	19 (25.0)
HT	21 (27.6)
Antihypertensive medications	
Beta-blocker	10 (13.2)
ACEi	12 (15.8)
Calcium channel blocker	1 (1.3)
Diuretic	4 (5.3)
Fasting period (hour)	15 (2–19)
Time of procedure (minute)	15 (5–43)
Propofol dose (mg)	165 ± 45
Fentanyl dose (µg)	76 ± 14

ACEi: Angiotensin-Converting Enzyme inhibitors, ASA: American Society of Anesthesiologists, BMI: Body Mass Index, CAD: Coronary Artery Disease, DM: Diabetes Mellitus, HT: Hypertension, and SD: Standard Deviation.

**Table 2:** The relationship between risk factors and the orthostatic hypotension group.

	Non-Hypotensive Group (n = 58)	Hypotensive Group (n = 18)	*p* Value
Age	53 (25–79)	55.5 (23–73)	0.922 ^a^
Sex			0.426 ^b^
Female	26 (44.8)	10 (55.6)	
Male	32 (55.2)	8 (44.4)	
BMI	27.7 ± 4.3	27.2 ± 5.8	0.756 ^c^
ASA status			0.523 ^d^
ASA 1	15 (25.9)	7 (38.9)	
ASA 2	37 (63.8)	10 (55.6)	
ASA 3	6 (10.3)	1 (5.6)	
CAD	7 (12.1)	3 (16.7)	0.693 ^d^
DM	15 (25.9)	4 (22.2)	1.000 ^d^
HT	14 (24.1)	7 (38.9)	0.240 ^d^
Antihypertensive medications			
Beta blocker	8 (13.8)	2 (11.1)	1.000 ^d^
ACEi	10 (17.2)	2 (11.1)	0.720 ^d^
Calcium channel blocker	-	1 (5.6)	0.237 ^d^
Diuretic	4 (6.9)	-	0.567 ^d^
Fasting time (hour)	15.5 (2–19)	14.5 (5–18)	0.640 ^a^
Time of procedure (minute)	15 (5–43)	15 (5–40)	0.752 ^a^
Hypotension Observed during procedure			0.077 ^d^
None	42 (72.4)	12 (66.7)	
Intraop	7 (12.1)	6 (33.3)	
Postop	8 (13.8)	-	
Intraop + Postop	1 (1.7)	-	
Vasopressor use			0.290 ^d^
None	46 (79.3)	15 (83.3)	
Intraop	4 (6.9)	3 (16.7)	
Postop	7 (12.1)	-	
Intraop + Postop	1 (1.7)	-	
Revovery time (minute)	12.9 ± 4.1	13.4 ± 3.8	0.46 ^c^

^a^ Mann-Whitney U test; ^b^ Pearson chi-square test; ^c^ Student’s *t*-test; ^d^ Fisher’s exact test. ACEi: Angiotensin-Converting Enzyme inhibitors, ASA: American Society of Anesthesiologists, BMI: Body Mass Index, CAD: Coronary Artery Disease, DM: Diabetes Mellitus, HT: Hypertension, and SD: Standard Deviation.

**Table 3:** Relationship between risk factors and symptoms of orthostatic intolerance.

	No Symptoms (n = 62)	With Symptoms (n = 14)	*p* Value
Age	54 ± 13.9	42.7 ± 15.4	0.009 ^a^
Sex			0.046 ^b^
Female	26 (41.9)	10 (71.4)	
Male	36 (58.1)	4 (28.6)	
BMI	28.1 (18.8–37.5)	24.1 (20.8–34.7)	0.062 ^d^
ASA status			0.543 ^c^
ASA 1	17 (27.4)	5 (35.7)	
ASA 2	38 (61.3)	9 (64.3)	
ASA 3	7 (11.3)	-	
CAD	8 (12.9)	2 (14.3)	1.000 ^c^
DM	15 (24.2)	4 (28.6)	0.740 ^c^
HT	20 (32.3)	1 (7.1)	0.095 ^c^
Antihypertensive medications			
Beta blocker	10 (16.1)	-	0.193 ^c^
ACEi	12 (19.4)	-	0.108 ^c^
Calcium channel blocker	1 (1.6)	-	1.000 ^c^
Diuretic	4 (6.5)	-	1.000 ^c^
Fasting time (hour)	16 (2–19)	14 (5–18)	0.220 ^d^
Time of procedure (minute)	15 (6–43)	15 (5–26)	0.455 ^d^
Vasopressor			0.272 ^c^
None	50 (80.6)	11 (78.6)	
Intraoperatif using	4 (6.5)	3 (21.4)	
Postoperatif using	7 (11.3)	-	
Intraop + postop	1 (1.6)	-	
Hypotension			0.913 ^c^
None	44 (71.0)	10 (71.4)	
Intraop	10 (16.2)	3 (21.4)	
Postop	7 (11.3)	1 (7.1)	
Intraop + postop	1 (1.6)	-	

^a^ Mann-Whitney U test; ^b^ Pearson chi-square test; ^c^ Student’s *t*-test; ^d^ Fisher’s exact test. ACEi: Angiotensin-Converting Enzyme inhibitors, ASA: American Society of Anesthesiologists, BMI: Body Mass Index, CAD: Coronary Artery Disease, DM: Diabetes Mellitus, HT: Hypertension, and SD: Standard Deviation.

**Table 4:** Comparison of preoperative blood pressure between patients with and without hypotension.

Hypotension Type	BP Type	Non Hypotensive Group	Hypotensive Group	*p* Value
Orthostatic hypotension	SBP	136.4 ± 20.7 mmHg	145.1 ± 25.9 mmHg	0.205
	DBP	83.9 ± 13.0 mmHg	92.9 ± 13.9 mmHg	0.022
Intraoperative hypotension	SBP	138.8 ± 20.6 mmHg	143.2 ± 31.6 mmHg	0.646
	DBP	87.1 ± 13.2 mmHg	84.2 ± 17.1 mmHg	0.581

BP: Blood Pressure, DBP: Diastolic Blood Pressure, and SBP: Systolic Blood Pressure.

## Data Availability

The datasets used and/or analyzed during the current study are available from the corresponding author on reasonable request.

## Abbreviations

The following abbreviations are used in this manuscript:

ACEi	Angiotensin-Converting Enzyme inhibitors
ARBs	Angiotensin Receptor Blockers
ASA	American Society of Anesthesiologists
BP	Blood Pressure
CAD	Coronary Artery Disease
CCBs	Calcium Channel Blockers
DBP	Diastolic Blood Pressure
DM	Diabetes Mellitus
HT	Hypertension
OH	Orthostatic Hypotension
OI	Orthostatic Intolerance
PACU	Post-Anesthesia Care Unit
POTS	Postural Orthostatic Tachycardia Syndrome
SBP	Systolic Blood Pressure
SD	Standard Deviation
